# Taking Action or Thinking About It? State Orientation and Rumination Are Correlated in Athletes

**DOI:** 10.3389/fpsyg.2019.00576

**Published:** 2019-03-26

**Authors:** Alena Kröhler, Stefan Berti

**Affiliations:** Department of Clinical Psychology and Neuropsychology, Institute for Psychology, Johannes Gutenberg-University Mainz, Mainz, Germany

**Keywords:** rumination, action control theory, state orientation, action orientation, failure-related behavioral adaptation, competitive sports, competition-related rumination, competitive athletes

## Abstract

Athletic performance in competitive sports relies heavily on the ability to cope effectively with stressful situations. In contrast, some athletes report that their thoughts revolve around the future or past and not around the actual demands during competitions. In those specific stressful situations, the lack of focus like an unintended fixation on repetitive cognitions can have fatal consequences with regard to the performance. Especially when competitors are close in their athletic capabilities, differences in effectively coping with stress and mental stability may decide about winning and losing. One established factor of performing effectively under pressure is the individual tendency to either focus on taking action (i.e., action orientation) or on focusing on the own emotions (i.e., state orientation). It is widely acknowledged that state-oriented athletes have disadvantages in performing under stress. Moreover, the action control theory claims that state orientation is related to ruminative cognitions, which itself is assumed to impair performance in the long term. We tested this hypothesis in 157 competitive athletes from different sports (including individual and team sports). Regression analysis demonstrates a substantial correlation of failure-related action orientation (i.e., state orientation) with different measures of rumination (including general, clinically relevant, and competition-related rumination). In addition, general (i.e., content independent) rumination also correlated substantially with a rumination scale adapted specifically to sports-related competition. These results suggest (1) that a sports and competition-related ruminative mechanism exists and (2) that ruminative cognitions are related to the cognitive basis of state orientation. While our study does not allow for a causal interpretation, it provides an additional approach to investigate mental factors underlying inter-individual differences in athletic performance under stress and pressure.

## Introduction

Competitive athletes distinguished themselves through conscious permanent acting under stressful conditions ascribable to their special environment, the participation in competitions, and the immediate consequences of their actions. In general, those situations require a high degree of immediate and long-term self-regulatory capabilities. Therefore, successful athletes were attributed to problem-focused, resilient coping-strategies (Henkel and Schneider, 2014). However, in the high demanding domain of competitive sports, inter-individual differences in the competence of adaption to stressful situations are observable and can have an enormous positive or negative impact on the athletic behavior and performance, especially throughout a competitive season or in the period of a training camp (Filho et al., 2013, 2015; Kölling et al., 2015). One theory that is well established in this context is the *action control theory* (Beckmann and Kossok, 2018), which was originally developed by Kuhl (1983, 1994a) and represents one of the most prominent theories in the context of volition. In essence, this theory assumes that regulation becomes necessary if conflicts between competing action tendencies occur (Beckmann and Kossok, 2018). Take, for instance, a soccer player who has to decide how to act after playing a bad pass during an attack: there might be different options for taking immediate action (like tackling an opponent to regain the ball or running back to support the defense) but it is also possible to focus on the lost (i.e., by cursing the conditions or the teammates or feeling guilty or incompetent). According to the theory of action control, in such situations, volitional processes are influenced by individual differences in action orientation. In detail, the theory distinguishes between action and state orientation, two extremes on a continuous scale describing the likelihood whether people respond with action-taking when situational demands are increasing. Action-oriented individuals distinguish themselves through solving problems intuitively in adverse conditions (e.g., bad weather, broken equipment, and poor field or arena conditions), rapid acting without excessively thinking about the source or the person responsible, and developing different possibilities to act in demanding situations (Kuhl and Kazén, 2003). They typically act in high demanding situations as efficiently or even better as in comparable relaxing situations (Koole and Jostmann, 2004; Koole et al., 2012). Athletes with an action orientation can also handle failures in high demanding situations more efficiently and draw the attention to forthcoming challenges.

In contrast, athletes with a tendency to state orientation are focused on their emotions and thoughts. An unintended fixation on the own situation is mostly the consequence, which is why they do not solve problems easily and refocus on the actual task (Kuhl and Kazén, 2003; Koole et al., 2012). Moreover, state-oriented athletes think a lot about their goals but fail to take immediate action. This behavior, therefore, could inhibit the readiness and the implementation of action (Dibbelt and Kuhl, 1994). Finally, in the theory of action orientation, ruminative cognitions are described as the most immediate and conscious consequence of a dispositional state orientation (Kuhl, 1994a).

A number of studies tested whether these hypothesized differences of action- and state-oriented individuals comply in the context of competitive sports. The previous findings pointed mainly in the same direction, suggesting disadvantages of state-oriented athletes compared to action-oriented athletes in different aspects that are relevant for athletic performance particularly in stressful situations. Two studies found differences in the level of action orientation and risk-taking behavior in the sense of measuring accuracy and time of the individual decision-making process (Stiensmeier-Pelster et al., 1989; Raab and Johnson, 2004). Further studies with different experimental paradigms showed differences in depletion of self-control resources (Gröpel et al., 2014) and in the efficiency of intention initiation (Kazén et al., 2008). This picture is generally supported by a review of Koole et al. (2005) who provided a summary of disruptive effects of stress on state-oriented individuals. In addition, Koole et al. (2005) described also potential positive effects of state orientation. In detail, state orientation can be adaptive (1) through external support, (2) in dangerous, unpredictable environments and (3) in interpersonal relationships. This applies to sports, too: for instance, Beckmann and Kazén (1994) reported inverse effects of state orientation in the sense of a positive consequence by maximum power. This shows that state orientation does not necessarily exhibit only negative effects on sport performance (see also Beckmann and Kossok, 2018, for a summary of advantages and disadvantages of the individual action orientation). However, the majority of studies demonstrated the disadvantage of state orientation in sports performance and the question arises whether this effect is related to the higher tendency of state-oriented persons to focus on thoughts and the assumed higher level of rumination (Kuhl, 1994a).

The effect of rumination on athletic behavior and performance has frequently been stated but was only rarely explicitly investigated. Only a few studies examined the mediating role of rumination indirectly in the process of athletic performance: one study with 305 competitive athletes examined the relationship between anger rumination and athlete aggression based on the Anger Rumination Scale (Maxwell, 2004). Results revealed a significant correlation between anger rumination and the athletes’ reported aggressive behavior. However, a direct relation of rumination and the athletes’ performance was not investigated. A study by Scott et al. (2002) confirmed a negative correlation between an overall measure of rumination (Scott-McIntosh Rumination Inventory; Scott and McIntosh, 1999) and a composite of measure of athletic performance of tennis players. The main limitation of this study was the small sample size (*N* = 10), which did not advocate a broad generalization. Roy et al. (2016) investigated the relation between rumination and performance in soccer and field hockey players using the Ruminative Response Scale (RRS; Treynor et al., 2003). They found an expertise effect mirrored in lower reflective rumination in athletes (professionals and nonprofessionals) compared with non-athletes. Furthermore, Roy et al. (2016) assumed that low scores on the RRS are associated with a longer career at a higher level in soccer players. Here, the level of expertise (professional vs. nonprofessional players) and the duration of a successful sports career defined the athletes’ performance. This suggests that at least success over the long term is correlated with rumination. However, a recent study also suggests that the achievement of short- and mid-term sports-specific goals is related to rumination (see Kröhler and Berti, 2017).

The aim of the study was to investigate the assumed relation between action orientation and rumination (see Kuhl, 1983, 1994a). As summarized above, the action control theory claims that rumination is an effect of a lower action orientation (i.e., higher state orientation) under stress. As both, the individual degree of action orientation as well as rumination, show associations with sports performance, it remains open whether a correlation of state orientation and rumination really exists in athletes. Therefore, we tested whether state orientation in the context of failure is correlated with rumination in general. The action control theory does not limit the theoretical claim to a specific situation, which implies that such a correlation should be observable in this highly selective population, too. In contrast, competitive athletes are highly trained in performing under pressure (i.e., in highly competitive situations) and, therefore, might exhibit competences in coping especially with potential or actual failures, which would result in increased stress in a normal population. From this point of view, athletes may not demonstrate a correlation between their levels of action orientation and rumination (i.e., on the trait level). However, even if athletes acquired specific competences in performing under stress, it remains open whether these competences are effective in general or in competitive situations only.

To date, different scales exists to measure action or state orientation either in general (Kuhl, 1994b) or in sports-related (Beckmann and Wenhold, 2009) context. Both measurements, the general Action Control Scale (ACS-90; Kuhl, 1994b; German Version: HAKEMP-90; Kuhl, 1990) and the sports-specific measure of action orientation in competitive sports (German: *Handlungsorientierung im Sport*; HOSP; Beckmann and Wenhold, 2009), consist of three subscales, namely (1) action orientation subsequent to failure scale (German: *Handlungsorientierung nach Misserfolg*; HOM), (2) prospective and decision-related action orientation scale (German: *Handlungsorientierung bei Entscheidungs- und Handlungsplanung*; HOP), and (3) action orientation during (successful) performance of activities (German: *Handlungsorientierung bei Tätigkeitsausführung*; HOT). Here we focus on the first subscale (HOM), which describes the capability to suppress failures and refocus on the following task immediately. With regard to rumination, we applied rumination questionnaires from three different contexts: clinically relevant rumination (RRQ, König, 2012), rumination in general (PTQ, Ehring et al., 2011), and competition-related rumination (modified from Krys et al., 2018).

We expect a relationship between HOM and rumination because the action control theory assumes that higher failure-related action orientation is associated with lower individual rumination. To test this hypothesis, we first conduct a correlational analysis. In addition, we investigate this relationship by applying linear robust regression analysis to quantify the potential association.

## Materials and Methods

### Subjects and Procedure

Within a period of 3 months (October–December 2016), athletes from different sports, including team sports as well as individual sports, participated in an online study voluntarily. We conducted the study in compliance with the Declaration of Helsinki (World Medical Association, 2013). The online instruction contained information about the nature and the procedure of the study and all participants gave consent before completing the questionnaires. The participants received no incentives for completion of the survey. For athletes under the age of 18, we obtained additional consent from the legally authorized representatives. The participants completed the action orientation subsequent to failure scale (HOM, Beckmann and Wenhold, 2009), the Perseverative Thinking Questionnaire (PTQ; Ehring et al., 2011), the Rumination scale from the Rumination-Reflection Questionnaire (RRQ; König, 2012) and a competition-related rumination scale (KSR-WK; modified from Krys et al., 2018). In addition to these, the participants filled out biographical and sports-related questions as well as other questionnaires, which were unrelated to the present study. We describe the utilized questionnaires below.

Overall, 210 athletes participated in our online survey. Criteria for selecting the subjects for our analysis were as follows: athletes between 15 and 30 years of age corresponding to the athletic high-performance age (Willimczik et al., 2006; Conzelmann, 2017) and a background in competitive sports. The final sample of athletes who met these criteria was 157 (female: *n* = 80, male: *n* = 77). Mean age was 21.57 years (*SD* = 3.63). The athletes averaged 10.00 h (*SD* = 5.60) of discipline-specific training in 4.46 training sessions (*SD* = 2.54) and 2.54 additional sessions (*SD* = 1.99; e.g., weight or athletic training) per week. The averaged participation in competitions per year was 13.40 (*SD* = 8.02). Besides, 32 athletes were already part of the junior national team and 16 athletes were part of the senior national team in their sports. Ten of these 48 athletes were in the junior as well as in the senior national team of their sports. According to the categorization of Beckmann and Wenhold (2009), the sample was assigned into six different sport categories and two performance levels (see Table 1). Depending on the organizational form of the disciplines (leagues or squads system), the sample was divided into two performance levels: performance level 1 includes all athletes belonging to highest to the third highest national level (comparable with A- to C-squad or First German Bundesliga as well as participations in German/European/World Championships or Olympic Games; *n* = 74). Performance level 2 includes all athletes belonging to the fourth highest or subjacent level (comparable with D-squad, Second German Bundesliga or below as well as participation in German Junior or regional championships; *n* = 83).

**Table 1:** Distribution of athletes separated in sport categories, gender and performance level.

Sport category	Performance level 1		Performance level 2		
	Female	Male	Female	Male	Sum
Ball sports-individual	1	1	3	2	7
Ball sports-team	7	5	13	23	48
Endurance sports-individual	24	25	19	15	83
Coordinative-compositional	6	2	5	0	13
Martial arts	0	2	1	2	5
Target focus	1	0	0	0	1
Sum	39	35	41	42	157

Examples: ball sports-individual = (table) tennis; ball sports-team = basketball, football; conditioning-individual = swimming, athletics; coordinative-compositional = rhythmic gymnastics, snowboard; martial arts = boxing, taekwondo; target focus = billiard, sport shooting.

### Measures

#### Failure-Related Action Orientation

We measured the failure-related action orientation with the German action orientation in sports questionnaire (HOSP, Beckmann and Wenhold, 2009). The HOSP is a standardized self-evaluation assessment with 36 items, consisting of three scales: action orientation subsequent to failure, decision-related action orientation, and action orientation during performance. Each scale consists of 12 items, which describe a particular situation. Each item has two alternative answers (A or B), one of which is indicative of action orientation and the other of state orientation. For the present study, only the action orientation subsequent to failure scale is relevant (HOM). The HOM measures the athletes’ ability to cope with failures and focus again on new demands. For athletes with lower values on this scale compared to those with higher values, it is difficult to cope with failures. They fight intensively with the setback, by what they may affect the execution of following tasks. For instance: “If I miss a clear chance of winning …” then A: “it sticks in my mind during the rest of the competition” (state orientation), or B: “I forget this failed attempt and concentrate on the next chance” (action orientation) [translations from the German original by AK].

Athletes earn one point for choosing the action-oriented answer. The sum of the action-oriented answers for each scale is between 0 and 12. The following applies: the higher the characteristics in the action orientation subsequent to failure scale, the higher the action orientation in this context.

#### Perseverative Thinking

The German version of the Perseverative Thinking Questionnaire (PTQ; Ehring et al., 2011) is a questionnaire independent from content for measuring repetitive negative thoughts. The PTQ consists of 15 items and is rated on a 5-point Likert scale, ranging from “0” (never) to “4” (almost always). In each case, three items correspond to a process characteristic of repetitive negative thinking, building one subscale: (1a) repetitive (e.g., “The same thoughts keep going through my mind again and again”), (1b) intrusive (e.g., “Thoughts come to my mind without me wanting them to”), (1c) difficult to disengage from (e.g., “I can’t stop dwelling on them”), (2) unproductive (e.g., “I keep asking myself questions without finding an answer”), (3) capturing mental capacity (e.g., “My thought prevent me from focusing on other things”; all items are taken from the English original).

The PTQ provides three scores for the particular subscales as well as a general PTQ score, which is the sum of the three subscales’ scores. Here, we report the general PTQ score. The internal consistency (Cronbach’s alpha: α) for the entire PTQ is α = 0.95 for the original and α = 0.94 for the present sample. Therefore, the analysis of the internal consistency in the present sample supports previous findings.

#### Rumination

Trapnell and Campbell (1999) developed the Rumination-Reflection Questionnaire (RRQ), for differentiating rumination and reflection, two relevant factors of private dispositional self-focus. The original version comprises 24 items in two scales: 12 items for rumination as well as for reflection. In the present study, we utilized the German version (König, 2012) and applied only the Rumination scale. This scale measures the self-attentiveness motivated by perceived threats, losses, or injustices to the self (Trapnell and Campbell, 1999). For instance, “Often I’m playing back over in my mind how I acted in a past situation.” Athletes rate the different statements on a 5-point Likert scale presenting the level of agreement ranging from “1” (strongly disagree) to “5” (strongly agree). The value of the items 6, 9, and 10 should be reversed. The individual test score can be calculated by adding up all values of the 12 items. Therefore, the scores are ranging between 12 and 60, and the higher the scores the higher the individual level of rumination. The internal consistency for the original sample is α = 0.90 (Trapnell and Campbell, 1999) and for the present sample α = 0.88.

#### Competition-Related Rumination

We used a questionnaire from Krys et al. (2018) for measuring the handling with difficulties in the context of a competition. The original version contains eight items according to learning-related difficulties during the preparation for a statistic exam (for instance, “I can’t stop thinking about learning-related problems”). The original questionnaire was developed in the way that the problem-related context is changeable. Therefore, we modified the items (Krys et al., 2018) to competition-related problems (KSR-WK); e.g., “I can’t stop thinking about competition-related problems.” Athletes respond on a 5-point Likert scale, ranging from “1” (does not apply at all) to “5” (fully applies).

The individual test score can be calculated by adding up all values of the eight items. Therefore, the scores are ranging between 8 and 40 and; again, the higher the scores the higher the individual level of rumination. The internal consistency for the original sample lies between α = 0.91 and α = 0.94 (Krys et al., 2018) and for the present sample α = 0.92.

### Data Analysis

We analyzed the relationship between failure-related action orientation and rumination by means of three single robust regression models. In doing so, failure-related action orientation represents the independent variable and the scales of PTQ, RRQ and KSR-WK represent the dependent variable for each single regression model. Beforehand, we checked the requirements for the application of regression analyses. We generated Q-Q plots for testing the assumption of normal distribution (Kabacoff, 2015), conducted analyses for testing the independence of the predictor variable including the standard errors (Durbin-Watson Test; Field et al., 2012) as well as the multi-collinearity of all used variables (VIF: variance inflation factor; Field et al., 2012). Except the normal distribution, all requirements for regression analyses were fulfilled. In addition, we analyzed outliers and influential cases using Cook’s distance, leverage values, and the proportion of co-variances (Field et al., 2012). While there were no outliers in the data, Cook’s distance revealed the existence of influential cases. These could have a considerable impact of the constant and the gradient of the regression model. We decided not to exclude the influential cases from further calculation. Instead, we applied robust regression analyses with a MM-estimation (a kind of maximum-likelihood estimation; Susanti and Pratiwi, 2014). This method uses a criterion, which is less vulnerable for influential cases. It has also a high breakdown value (general measurement of the proportion of influential cases, which are edited before influencing the regression model; Susanti and Pratiwi, 2014). Hence, the robust method is more appropriate in calculating regressions. Moreover, it is possible to conduct the regression analyses with all observed cases by restricting the effect of influential cases *via* Cook’s distance and high leverage values at the same time.

## Results

Table 2 sums up the descriptive statistics among the study variables for all competitive athletes (*N* = 157).

**Table 2:** Descriptive statistics among the study variables for all competitive athletes (*N* = 157).

	*M*	*SD*	*SE*	Median	Skew	Kurtosis	95% CI
HOM	6.35	3.12	0.25	6	−.0.09	−0.83	[5.86, 6.84]
PTQ	25.5	10.66	0.85	24	0.52	0.52	[23.82, 27.18]
RRQ	37.07	9.02	0.72	38	−0.06	−0.39	[35.65, 38.49]
KSR-WK	19.37	7.11	0.57	19	0.51	−0.36	[18.25, 20.49]

*M*, mean; *SD*, standard deviation; *SE*, standard error of mean; 95% CI, 95% confidence intervals.

**Figure 1:**
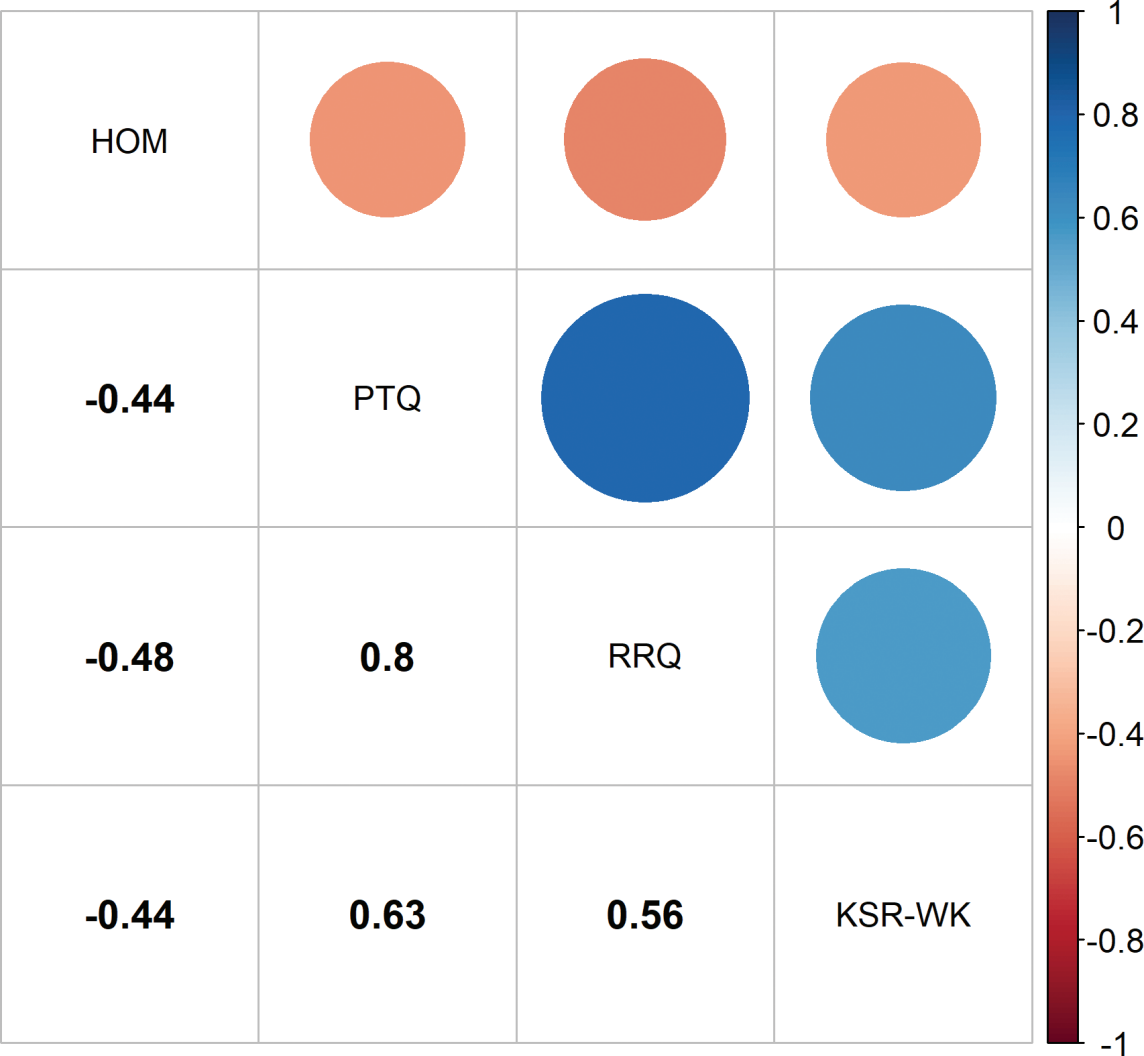
Summary of the Pearson correlations coefficients (*r*) between action orientation subsequent to failure scale and three rumination scales. The diagonal depicts the individual scales used in this study. The arrays under the diagonal depict the correlational coefficients of the particular scales (all *p*’s < 0.05 [Holm corrected *p*’s for multiple comparisons]; all *df*’s = 155). The arrays above the diagonal illustrate these values in a symbolic way with the size of the circles specifying the extent of the parameter value (values between 0 and 1) and the color of the circles depicting the direction of the parameter value (positive or negative; see color scale at the right).

**Figure 2:**
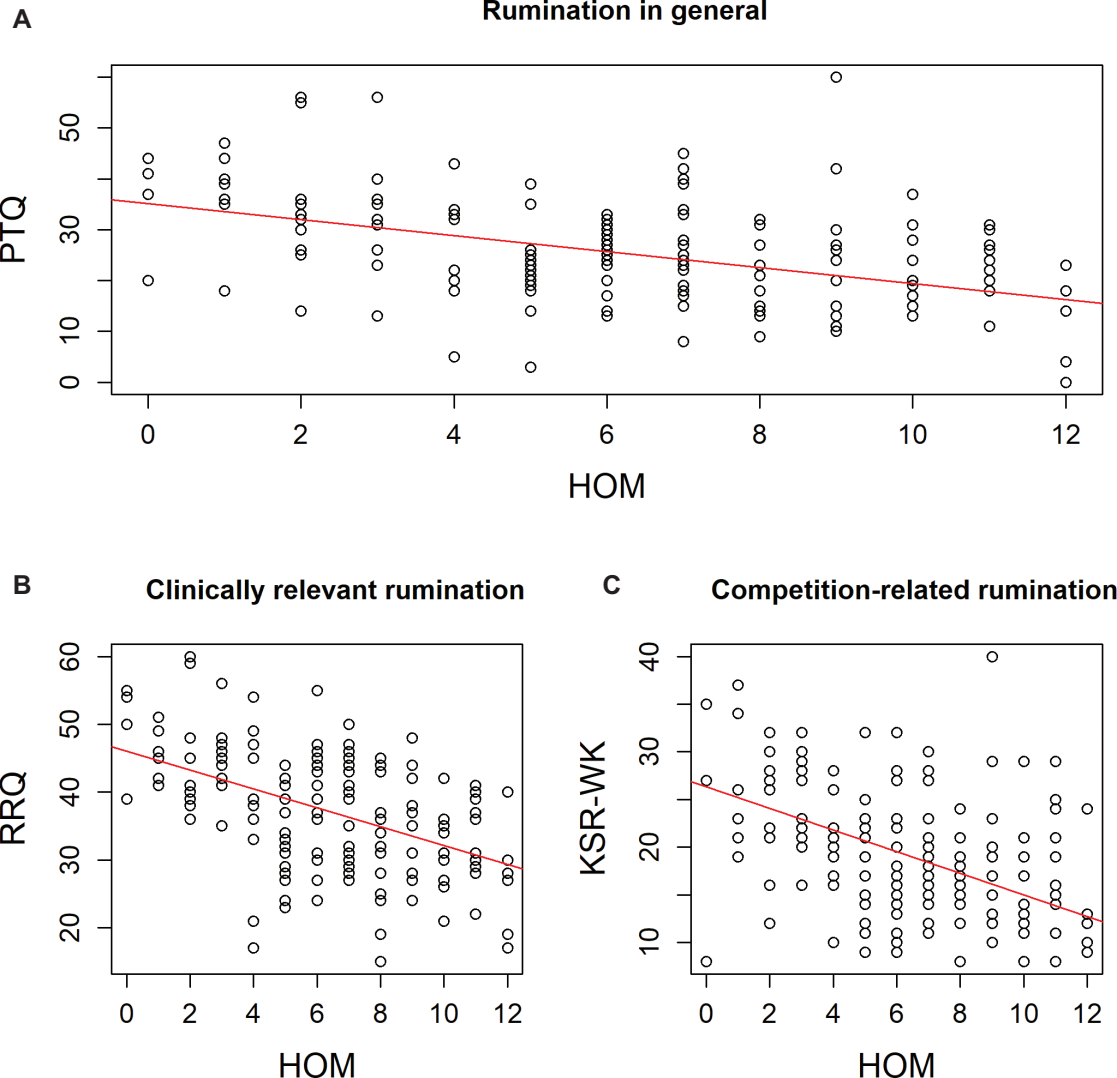
Scatterplots of failure-related action orientation (HOM; range 0–12) and rumination (*N* = 157). In detail, HOM is plotted with **(A)** rumination in general (PTQ; range 0–60), **(B)** clinically relevant rumination (RRQ; range 12–60), and **(C)** competition-related rumination (KSR-WK; range 8–40). The regression lines are based on robust linear regressions of HOM with the respective rumination measure (for details see text).

The results of the correlational analysis revealed significant associations between failure-related action orientation (HOM) and the three rumination scales (PTQ, RRQ, KSR-WK; *p* < 0.05; alpha corrected with Holm, see Figure 1). Figure 2 demonstrates that there is a substantial, inter-individual variation in all three rumination measures in the participating athletes but the robust regression analyses indicated that failure-related action orientation significantly predicts rumination (see Table 3). In addition, we determined the power of our regression analyses (G*Power 3.1.; Faul et al., 2009) with the sample size *N* = 157, an alpha level α = 0.001, and the obtained medium effect sizes; this analyses revealed a power of 0.9998 for the PTQ regression (*f* ^2^ = 0.30), 0.9997 for the RRQ regression (*f* ^2^ = 0.28), and 0.9999 for the KSR-WK regression (*f* ^2^ = 0.32).

**Table 3:** Characteristics of the single robust regression analyses with failure-related action orientation as predictor and three rumination scales as criterion.

Criterion	Predictor	*B*	*SE B*	β	*p*	R2adj
PTQ	HOM	−1.57	0.24	−0.46	<0.001	0.23
RRQ	HOM	−1.25	0.17	−0.48	<0.001	0.22
KSR-WK	HOM	−1.13	0.19	−0.50	<0.001	0.24

## Discussion

Our study demonstrates in competitive athletes a direct relationship of rumination and action orientation after failure. This supports the claims of the action control theory (Kuhl, 1983, 1994a) and does suggest an expansion to context-specific situations (here, competitive sports). The correlational analyses show middle to strong association between failure-related action orientation and all three rumination scales. Findings of the regression analyses support our hypothesis, in the way that failure-related action orientation is a significant predictor for rumination as reflected in a general, a clinically oriented as well as a competition-related measure. It is worth noting that the explained variance in the regression analyses is about 20% for all three rumination measures. On the one hand, this indicates that a general “ruminative” factor is shared by these different measures and that this aspect of rumination indeed is linked to action control (as assumed by Kuhl, 1983, 1994a). On the other hand, this leaves a lot of variability in the data, which is not explained by individual level of action orientation. While this might be attributed to different specific characteristics of rumination captured by these particular scales (e.g., well-being in a clinical context vs. negative outcome in the context of a competition), this may also suggest that neither of the applied rumination scales is already suitable for a competitive athletes’ population. However, the correlation between the competition-related rumination (KSR-WK) and the general rumination scale (PTQ) indicates that the applied variant of the KSR-WK (Krys et al., 2018) does tap rumination in a specific context. Both variables share a common variance of nearly 40%, indicating that besides the general factor an independent competitive specific factor emerged.

In general, our findings are in line with previous studies (e.g., Beckmann and Trux, 1991; Beckmann and Kazén, 1994) and indicated that rumination might be a relevant factor for individual requirements in competitive sports. As the aim of coaches in competitive sports is to help athletes to gain their optimal performance, additional information about athletes’ disposition in relevant mental factors may allow coaches to adapt to the individual needs of their athletes. For instance, in team sports, the knowledge about the athletes’ level of action or state orientation could be beneficial when selecting playing positions or deciding ball allocation strategies depending on different gaming situations. This is suggested by a study of Beckmann and Trux (1991), who showed that key players in high-performance professional sports tend to be state oriented rather than action oriented, whereas the strikers were mainly action oriented. Beckmann and Kossok (2018) suggested applying the knowledge of athletes’ dispositions in order to selectively introduce them to different disciplines or positions in which their personal dispositions might promise particular success. Therefore, from the perspective of applied sports psychology, the concept of rumination offers a number of potential applications. For instance, athletes and coaches typically perceived ruminative thoughts (especially with negative content) as a limiting factor for gaining high performance. One aim could be to identify athletes with a predisposition toward extensive rumination especially in younger ages. It is also promising to support young talents in their ability to control repetitive disruptive thoughts, because many athletes perform suboptimally in pressure situations despite a high motivation to succeed (Baumeister and Showers, 1986). Well-known moderators for suboptimal performance in pressure situations (choking under pressure) are among others trait anxiety, reinvestment, and perfectionism, which are all closely related to rumination (Flett et al., 2002; Nolen-Hoeksema et al., 2008; Kinrade et al., 2010). To this end, early identification of the individual dispositional rumination at the beginning of a sports career might enable a more effective support by application of treatments to avoid rumination (and thereby potential stress) in the long run. For instance, Roy et al. (2016) report a relationship between a successful sports career and a low reflective rumination style. Existing therapeutic interventions related to rumination (see Broderick, 2005; Nolen-Hoeksema et al., 2008; Watkins, 2008; Van Aalderen et al., 2012; Querstret and Cropley, 2013) therefore, might be adapted to the non-clinical group of athletes on the one hand. On the other hand, recent studies (Birrer et al., 2012; Mosewich et al., 2013; Josefsson et al., 2017) deal with rumination-related interventions in competitive sports. Contents of these interventions focused mainly on self-compassion (Mosewich et al., 2013) and mindfulness (Josefsson et al., 2017). Results already show an improved regulation of maladaptive thoughts, feelings, and behavior and therefore provide a promising approach for future research.

### Study Limitations and Future Directions

Our study revealed correlations between failure-related action orientation and rumination in three different contexts in competitive athletes. However, the time of survey was far away from the actual competition or a special failure-related situation. Therefore, we only received information about individual traits and cannot rule out that the obtained correlations are modulated by the experience of an upcoming or actual competition. This limits also our current understanding of whether the action orientation and rumination link is more of a trait or state. One future direction is to survey athletes immediately after the competition or a failure-related situation. For instance, ambulatory assessment with an event-based design could provide a promising approach: within a defined period, athletes could complete a short questionnaire related to action orientation and rumination immediately after the experience. This could also serve as an interesting starting point in gaining more information about differences in individual action orientation and the consequences of it. An open question is whether there is a direct link between failure-related action orientation and individual rumination. Due to the cross-sectional design (only one measurement), the results are only correlational in nature. In other words, additional research is required to investigate whether the relation of failure-related action orientation and rumination is replicable and generalizable in a broader population.

## Conclusion

The action control theory by Kuhl (1983, 1994a) claimed a link of rumination and state orientation and, therefore, provides a theoretical framework for examination of the negative effect of both on athletic performance. Here we demonstrate that this hypothesis in general holds in a very specialized population, namely competitive athletes. This suggests that both, the action control theory and the theoretical considerations related to rumination may offer further routes of investigating the nature of individual performance variations in athletes under stress, e.g., in the context of a competition.

## Data Availability

The datasets generated for this study are available on request to the corresponding author.

## Ethics Statement

The study was conducted in accordance with the Declaration of Helsinki and the Ethical Code of the German Society for Psychology (DGPs) and all subjects gave written informed consent prior to their participation in the study. In the case of our selection of participants (all healthy persons) and our study design the local ethics committee at the Institute for Psychology do not ask for an approvement.

## Author Contributions

AK and SB contributed with the conception and design of the study. AK collected the data and performed the statistical analysis, and AK and SB wrote the manuscript.

### Conflict of Interest Statement

The authors declare that the research was conducted in the absence of any commercial or financial relationships that could be construed as a potential conflict of interest.
